# Cancer Stem Cells in Pediatric Sarcomas

**DOI:** 10.3389/fonc.2013.00168

**Published:** 2013-06-27

**Authors:** Filemon S. Dela Cruz

**Affiliations:** ^1^Division of Pediatric Oncology, Department of Pediatrics, Columbia University Medical Center, New York, NY, USA

**Keywords:** sarcoma, cancer stem cell, osteosarcoma, Ewing sarcoma, rhabdomyosarcoma, aldehyde dehydrogenase, side population, sphere formation

## Abstract

Sarcomas represent a clinically and biologically diverse group of malignant connective tissue tumors. Despite aggressive conventional therapy, a large proportion of sarcoma patients experience disease recurrence which will ultimately result in mortality. The presence of a unique population of cells, referred to as cancer stem cells (CSCs), have been proposed to be responsible for refractory responses to current chemotherapies as well underlying the basis for metastasis and relapse of disease – clinical corollaries to what has been termed the CSC hypothesis. The presence of CSCs have been suggested in a variety of hematologic and solid malignancies, and only more recently in sarcomas. Based on our current understanding of normal stem cell biology and evidence obtained from the study of malignant hematopoietic and solid tumors, researchers have identified candidate cell surface markers (CD133, CD117, Stro-1), biochemical markers (aldehyde dehydrogenase activity), and cytological characteristics (side population and spherical colony formation) that may identify putative sarcoma CSCs. In this review, we explore the current state of evidence that may suggest the existence of sarcoma CSCs. We present research in osteosarcoma, the Ewing’s sarcoma family of tumors, rhabdomyosarcoma, as well as other sarcoma subtypes to describe commonly used molecular and biochemical markers, as well as techniques, used in the identification, isolation, and characterization of candidate sarcoma CSCs. We will also discuss the current controversies and challenges that face research in sarcoma CSC.

## Introduction

Sarcomas are a biologically and clinically heterogeneous group of malignant connective tissue tumors originating from mesenchymal or ectodermal tissue. Sarcomas clinically span the entire age spectrum with an annual incidence ranging from 1 to 6 per 100,000 persons, and comprising 1.5% of all malignant tumors in adults and up to 8% in children. The categorization of different sarcoma types have classically been based upon the histological resemblance of a given sarcoma subtype to the adult tissue which it most closely resembles. There are currently over 50 sarcoma subtypes described underscoring the clinical and biologic diversity of this group of malignant cancers. Despite conventional multi-modality treatment approaches (surgery, radiation therapy, and chemotherapy), patients diagnosed with sarcoma experience disproportionally higher rates of morbidity and mortality compared to patients with other cancers. Long term survival rates average ∼50% in adults and up to 70% in children, but a significant proportion suffer recurrence and dissemination of disease despite aggressive therapy with subsequent rates of survival no higher than 20%. Investigations into the biology of cancer resistance to therapy and cancer relapses despite initial remission of disease have resulted in the development of a cell model that may account for observed cancer treatment failures and disease recurrences – the cancer stem cell (CSC) hypothesis.

In this review, the CSC hypothesis is discussed in terms of how it may contribute to investigations exploring the origins of sarcomas. We will also discuss methodologies utilized to characterize and isolate so-called sarcoma CSCs and evaluate the evidence to support the existence of sarcoma CSCs in different sarcoma subtypes.

## Cancer Stem Cell Hypothesis

Malignant tumors, like the normal tissues from which they arise, are composed of diverse cell populations. Within any given normal tissue reside a subpopulation of cells with the distinct ability to self-renew and are imbued with the ability to give rise to more differentiated progenitor cells – the tissue stem cell. Similarly, a malignant tumor is composed of a heterogeneous population of cells with different degrees of differentiation, proliferation, and tumorigenic potential. A subset of these cells, termed the CSC, possesses the ability to self-renew and generate the diversity of cancer cells that constitute the malignant tumor reminiscent of the tissue stem cell. The hypothesis that CSCs lie at the apex of a cellular hierarchy wherein the CSC gives rise to distinct populations of tumorigenic and non-tumorigenic cells has evolved over the past decade and has uncovered complex models that seek to account for the phenotypic and functional heterogeneity of tumors (Magee et al., 2012).

In order to facilitate and enable the comparison and translation of data between different investigators working on cells derived from similar or divergent tissues, a consensus definition of CSCs will be essential. One definition proposed for CSCs is a cell contained within a tumor with the ability to: (1) self-renew, and (2) possesses the ability to generate the heterogeneous cell lineages which will constitute a tumor (Clarke et al., 2006). Thus, reminiscent of the self-renewal properties of a tissue stem cell, CSCs can undergo self-renewal through symmetric division to give rise to two identical daughter cells which maintain the parental phenotype and capacity for self-renewal, or can alternatively undergo asymmetric division to give rise to a self-renewing daughter cell and a second more differentiated cell (transit amplifying cell). It is further division and subsequent lineage commitment and differentiation of this latter type of cell that ultimately comprises the tumor bulk.

The origin of CSCs continues to be an area of considerable uncertainty and debate in sarcomas. The term “CSC” has been interpreted to imply that the CSC may originate from normal tissue stem cells that acquire genetic and epigenetic changes conferring a malignant, tumorigenic phenotype (Nowell, 1976; Clarke et al., 2006; Baylin and Jones, 2011). However, it is important to note that even within a cellular hierarchy, CSCs may arise from either tissue stem cells or more differentiated cells that have acquired stem cell-like properties during malignant transformation. The cells that comprise a given tumor may then undergo a process of clonal evolution that gives rise to different subpopulations of cells with variable biologic properties as a result of selection pressure influenced by the genetic and epigenetic changes acquired by the parental CSC. Although this view represents one possible hypothesis for the origin of the CSC and the development of tumor heterogeneity, this model has neither been definitively established nor does it necessarily apply to all tumors. Indeed alternative CSC models have described the importance of the tumor microenvironment in influencing the development of heterogeneous populations of CSC-derived tumor cells (Polyak et al., 2009; Charles et al., 2010; Bissell and Hines, 2011). Furthermore, the previously held understanding that the CSC is a rare subset of cells within a given tumor and that the development of tumorigenic and non-tumorigenic subpopulations of cells from a parental CSC is unidirectional has been recently revised (Quintana et al., 2010; Chaffer et al., 2011; Gupta et al., 2011). Evolution of the CSC hypothesis which incorporates the tumor microenvironmental influences and the potential for bidirectional interconversion of tumor cells between tumorigenic and non-tumorigenic phenotypes have added to the challenge in identifying a CSC population in sarcomas and will require improved understanding into the origin of the numerous sarcoma subtypes and the application of sophisticated techniques to identify, isolate, and characterize putative sarcoma CSCs (Trucco and Loeb, 2012).

## Isolation and Characterization of Sarcoma Cancer Stem Cells

Work involving the isolation and characterization of tissue stem cells has resulted in the identification of biologic markers and the development of techniques that have also been employed to isolate and characterize CSCs. In this section, we will review the molecular markers and techniques that enable investigators to identify, isolate, and characterize putative sarcoma CSCs (summarized in Table 1).

**Table 1:** **Methodologies for characterizing sarcoma stem cells**.

Sarcoma subtype	Stem cell markers and assays
Osteosarcoma	CD133
	CD117/Stro-1
	Side population/dye efflux
	ALDH
	Sphere formation
Ewing’s sarcoma family of tumors	CD133
	ALDH
	Side population/dye efflux
	Sphere formation
Rhabdomyosarcoma	CD133
	Sphere formation
Synovial sarcoma	CD133
	Side population/dye efflux
Liposarcoma	CD133
	ALDH
Fibrosarcoma	ALDH
Chondrosarcoma	CD133
Malignant fibrous histiocytoma (MFH; pleomorphic undifferentiated sarcoma)	Side population/dye efflux

### Descriptive cancer stem cell assays

#### Cell surface markers

The identification of cell surface markers that are specific to CSCs would be invaluable to the prospective identification and isolation of CSCs particularly since CSCs may sometimes, but not always, constitute a minute subpopulation of cells within a given tumor. Important features of a CSC cell surface markers would have to not only be specific to the CSC, but also should discriminate the CSC from non-CSC cells within the tumor and ideally not expressed by non-transformed, normal tissue cells. One of the first examples of a specific CSC cell surface marker was identified in acute myeloid leukemia (AML) cells (Lapidot et al., 1994). Subsequent work by numerous investigators have identified several other candidate CSC cell surface markers (e.g., CD34, CD44, CD90, CD117, CD133, CD20) in a variety of different solid tumors including brain, breast, prostate, melanoma, colon, lung, liver, and sarcomas (Al-Hajj et al., 2003; Singh et al., 2003; Collins et al., 2005; Fang et al., 2005; Eramo et al., 2008; Tirino et al., 2008).

A growing body of literature has described the cell surface marker CD133 as a putative CSC surface marker. CD133 is five-transmembrane domain protein encoded by the *PROM1* gene which was first recognized as a novel antigen on CD34+ progenitor hematopoietic stem cells. Subsequent work revealed that CD133 was also expressed on other normal human cells and tissues including brain, kidney, prostate, pancreas, liver, colon, stomach, uterus, and mammary glands (Mizrak et al., 2008; Wu and Wu, 2009). Research in brain tumors, followed by subsequent work in other solid tumors, revealed that the CD133+ subpopulation of cells were capable of recapitulating the parental tumor when the CD133+ cells were transplanted into immunodeficient mice (Singh et al., 2003, 2004; Suetsugu et al., 2006; Hermann et al., 2007; Klein et al., 2007; Monzani et al., 2007; Ricci-Vitiani et al., 2007; Eramo et al., 2008). These results suggest that CD133 may be useful for the identification of sarcoma CSCs. However, work in colon and brain cancers has revealed that CD133− cells were also able to give rise to tumors suggesting that CD133 positivity as a specific marker for CSCs may not be valid for all solid tumor or sarcoma subtypes (Ogden et al., 2008; Shmelkov et al., 2008; Wang et al., 2008). Prospective validation of CD133 positivity as a marker for sarcoma CSCs in other sarcoma subtypes would need to be performed in the future and perhaps be combined with other assays for the identification and isolation of putative sarcoma CSCs.

#### Stem cell gene expression

The pluripotent and self-renewal properties of embryonic stem cells and the derived tissue stem cells are maintained by the expression of a multitude of genes that have been collectively termed “stem cell genes.” Interestingly, a significant number of these stem cell-associated genes have also been implicated in tumorigenesis and has led investigators to further explore the role of stem cell-associated genes in malignant cell transformation. In studies of CSCs, demonstration of stem cell-associated gene expression has been used as evidence to support the identification of cells with stem cell-like properties (i.e., putative CSCs) in conjunction with other cell surface markers or functional assays. However, it is important to note that expression of any or all of the stem cell-associated genes is not sufficient for the identification of sarcoma CSCs as these genes are expressed by normal stem and committed progenitor cells. It is believed that re-expression of stem cell-associated genes by cancer cells may simply reflect the malignant transformation of cells and not necessarily unique to the CSC.

#### Side population and dye efflux exclusion

One of the characteristic features of CSCs is its inherent or acquired resistance to cytotoxic chemotherapy agents. One explanation for the relative chemoresistant properties of CSCs involves the overexpression of ABC (ATP-binding cassette) multidrug efflux transporters. Capitalizing on this feature of CSCs, several investigators have utilized a technique involving the use of fluorescent DNA-binding dyes Hoechst 33342 or Rhodamine 123 to identify putative CSCs through fluorescence-activated cell sorting (FACS) (Moserle et al., 2008; Fukuda et al., 2009; Song et al., 2010; Tirino et al., 2012). Expression of ABC transporters on CSCs result in the active efflux of these fluorescent dyes allowing CSCs to be identified as a “side population” (SP) of cells by flow cytometry. Similar to the isolation and characterization of CD133+ cells, SP cells from a variety of sarcomas have been isolated by FACS and shown to be capable of initiating tumor formation in immunodeficient mice as well as self-renewal properties as evidenced by serial transplantation experiments (Wu et al., 2007; Tirino et al., 2008; Murase et al., 2009). In contrast, non-SP cells were generally less capable of initiating tumors. One drawback of the dye exclusion technique to identify putative sarcoma CSCs is the inherent toxicity associated with the dyes used. Hence, cells with the ability to efflux the dye have a survival advantage over cells that are less able to efflux the dye and that the property of dye exclusion may not necessarily be shared by all sarcoma CSCs. Indeed, non-SP cells have been evaluated and shown to possess tumor-initiating and propagating properties including in sarcomas, albeit requiring larger quantities of tumor inocula, suggesting that dye exclusion is not an essential feature of sarcoma CSCs and that use of this technique for the isolation and characterization of sarcoma CSCs be done only in conjunction with other techniques and/or CSC-specific markers (Machalinski et al., 1998; Wu et al., 2007; Broadley et al., 2011).

#### Aldehyde dehydrogenase

Chemotherapy resistance, as previously mentioned, is a characteristic feature of CSCs. In addition to the overexpression of drug efflux transporters, CSCs can also upregulate the expression of detoxification enzymes as an alternative mechanism for chemotherapy resistance. Aldehyde dehydrogenase (ALDH) catalyzes the oxidation of intracellular aldehydes and biogenic amines and has also been described to play an important role in drug detoxification in a variety of normal and malignant cells. Work in leukemia cells lines, as well as in normal hematopoietic and neural stem progenitor cells, have revealed significant expression of ALDH up to 200-fold (Russo and Hilton, 1988; Chute et al., 2006). In addition to its detoxifying effects, ALDH may also be involved in regulating stem cell differentiation in hematopoietic stem cells through retinoid-mediated signaling (Muramoto et al., 2010).

Since progenitor stem cells can be characterized by elevated intracellular ALDH activity, investigators have examined if high ALDH activity could identify CSC populations (Awad et al., 2010; Ma and Allan, 2011). High levels of ALDH activity are indeed observed in CSC subpopulations in several solid tumors including breast, lung, liver, colon, as well as sarcomas. The fluorescent reagent (ALDEFLUOR^®^) commonly utilized in identifying ALDH^High^ cell sub-populations functions primarily as a substrate for the ALDH 1A1 and 3A1 isoforms. It remains to be determined if other ALDH isoforms are equally present and expressed in sarcoma CSCs and if alternate ALDH isoforms can be measured utilizing this technique.

### Functional cancer stem cell assays

#### Serial transplantation

The CSC is defined by its capacity to self-renew through symmetric division and recapitulate the heterogeneous lineages of cells through asymmetric cell division to yield more differentiated cells that characterize a given tumor. Given this definition of the CSC, the identification of a CSC relies on a functional assay whereby a candidate CSC is able to generate a tumor with the same heterogeneous cellular constituents of the original tumor from which the CSC was originally isolated. The tumor initiating and propagating properties of putative CSCs can be accomplished through serial transplantation of cells in immunodeficient mice – the current gold standard used in the identification and characterization of candidate CSCs.

In general, serial transplantation experiments involve the isolation of candidate CSCs through the use of selective molecular markers, followed by the subcutaneous or intravenous injection of cancer cells into immunodeficient mice (Baiocchi et al., 2010; Tirino et al., 2012). If the inoculated cells are able to give rise to tumors reminiscent of the parental tumor phenotype, the inoculated cells are then believed to contain CSCs. In comparison to non-selected cell populations or cell populations not positive for the cell marker of interest, the CSC cell population often require significantly lower cell inocula to initiate tumor formation in immunodeficient mice. However, there is currently no standard as to what strain of immunodeficient mouse to use for serial transplantation experiments. This point is of importance as the immune status of recipient mice have been shown to be important for the propagation of certain tumors (Chang et al., 2001; Quintana et al., 2008).

#### Sphere-formation assay

The ability of cells to form spherical colonies when grown under non-adherent conditions has been used to identify tissue stem cells and consequently CSCs. The ability of tissue stem cells and CSCs to form spheres was first described from studies involving the identification of neural stem cells which formed free-floating neurospheres after dissociation of brain tissue and culture in serum-free medium containing epidermal growth factor (Reynolds and Weiss, 1992; Pastrana et al., 2011). Similar techniques have been employed in the propagation of CSCs involving the growth of single cell suspensions of sarcoma cells grown in serum-free conditions to ultimately yield sarcospheres (Gibbs et al., 2005; Fujii et al., 2009; Tirino et al., 2011). However, sphere formation has also been shown to arise from non-stem cell populations (Pastrana et al., 2011). Hence, the ability to form spheres in culture may not necessarily identify sarcoma CSCs but rather reflect the response of cells when removed from their normal tissue microenvironment or niche. Furthermore, sphere-formation assays may fail to identify quiescent CSCs as the success of sphere-formation relies on an expanding cell population that is either poised to proliferate or are actively dividing.

## Sarcoma Stem Cells

The identification and characterization of CSCs from malignant solid tumors, such as breast, skin, brain, or colon, have prompted the search for this elusive population of cells in sarcomas. Adopting the techniques just described and developed in the investigation of normal and malignant stem cells, we now examine the current evidence suggesting the existence of CSCs in sarcomas.

### Osteosarcoma

Osteosarcomas comprise the most common malignant tumor of bone in children and adolescents. Therapy incorporating multi-agent cytotoxic chemotherapy and surgery has resulted in cure rates of 60–70% in the localized disease setting (Bacci et al., 1993). However, ∼15% of patients present with disseminated or metastatic disease at initial presentation and have poor survival rates of<20% despite aggressive multi-modal therapy (Harris et al., 1998). Given that one of feature of a CSC is its inherent resistance to cytotoxic chemotherapy drugs, the identification and direct targeting of sarcoma CSCs in osteosarcoma may improve the survival outcomes for all osteosarcoma patients, particularly those with metastatic disease.

One of the first reports of cells derived from osteosarcoma with stem cell-like properties was a study by Tsuchida et al. (2008) who showed that a SP of cells could be isolated by exposure of the HOS osteosarcoma cell line to the chemotherapy agent cisplatin. Although the cisplatin-treated SP cells demonstrated several CSC features including expression of stem cell-associated genes, colony growth, and tumor formation, SP cells from untreated cells failed to form tumors suggesting that the SP population in treated cells may have undergone clonal selection and enrichment for cells with increased tumorigenicity, but not necessarily identification of a sarcoma CSC.

A subsequent study by Tirino et al. (2008) evaluated the utility of CD133 expression to identify and isolate a subpopulation of cells with CSC properties in osteosarcoma cell lines. Examination of osteosarcoma cell lines identified a subpopulation of CD133+ cells which exhibited self-renewal properties, higher proliferative rates, spherical colony formation, and expression of the stem cell-associated gene *OCT3/4*. However, the tumorigenic potential of CD133+ population was not evaluated through *in vivo* xenotransplant assays. A follow-up study by the same group evaluated 21 patient-derived sarcoma tissues which showed variable expression of CD133 (Tirino et al., 2011). Relatively high CD133 expression was observed in 2 osteosarcoma tissues examined which were successfully maintained in *in vitro* cell culture. However, the CD133+ fraction from both osteosarcoma cell lines were incapable of tumor formation when injected into non-obese diabetic (NOD)/severe combined immunodeficiency (SCID) mice which leads one to question the validity of CD133 expression in identifying a tumorigenic CSC population in osteosarcoma.

Other cell surface markers have been identified which appear to more selectively enrich for cells with tumor-initiating properties. Adhikari and colleagues examined the expression of two mesenchymal stem cell markers, CD117 and Stro-1, and discovered that mouse and human osteosarcoma cell lines positive for both CD117 and Stro-1 (DP or double positive cells) readily formed spherical colonies, more readily formed tumors in a nude mouse model in comparison to cells negative for both CD117 and Stro-1 (DN or double negative cells), had higher metastatic potential in an orthotopic NOD/SCID mouse model, exhibited increased chemoresistance when exposed to the chemotherapy agent doxorubicin, retained multipotent differentiation ability, and able to reconstitute DP and DN populations upon serial transplantation (Adhikari et al., 2010). This study has been the first to specifically identify a subpopulation of osteosarcoma cells with the hallmark features of a CSC: self-renewal capacity and the ability to reconstitute the original cellular makeup of a tumor or cell population upon serial transplantation. Validation of the stem cell-like properties of the DP population of cells in a broader panel of primary, patient-derived osteosarcoma cells would further strengthen the utility of CD117 and Stro-1 marker expression as specific markers for the identification, isolation, and therapeutic targeting of this unique population in osteosarcoma.

Analysis of SP cells, for the presence of sarcoma CSCs have also yielded cells exhibiting stem cell features. Murase et al. (2009) examined osteosarcoma cell lines and a malignant fibrous histiocytoma cell line and found that the MFH and only one of seven osteosarcoma cell lines had an identifiable SP fraction. Moreover, only the SP cells derived from the MFH cell line was able to form tumors in NOD/SCID mice, whereas SP cells from the osteosarcoma cell line could not. Interestingly, it has been recently shown that blockade of developmentally important signaling pathways, specifically hedgehog and Notch, in the MFH SP fraction abrogated its proliferative and tumorigenic capacities potentially implicating these pathways in the process of stem cell renewal and presenting a rationale for future therapeutic targeting (Wang et al., 2012). In contrast, Yang et al. (2010) derived SP cells from primary osteosarcoma cell lines and found that SP, as well as non-SP cells, were capable of tumor formation thus questioning the validity of SP cells as a specific CSC marker. These results underscore the importance of utilizing alternative assays and cell surface markers for the identification and isolation of putative sarcoma CSCs as SP-based isolation does not appear to be sufficient and selective for the enrichment of sarcoma CSCs. A lack of specific criteria and guidelines for the delineation of the SP fraction has likely contributed in the disparate results described. Alternatively, the inconsistent results from studies evaluating the SP cell fraction or various candidate CSC cell surface markers may reflect the cellular heterogeneity of osteosarcoma tumors. Furthermore, the identity of the CSC fraction in osteosarcoma may consist of a dynamic population of cells that may modulate its immunophenotype in response to microenvironmental cues and, thus, differ among cell lines and patients – a phenomenon described in other solid tumors (Quintana et al., 2010; Chaffer et al., 2011; Gupta et al., 2011). The ability to track individual cells within the tumor bulk may help to clarify these issues and help to identify CSCs in osteosarcoma.

Lineage tracking of cells offers a novel means by which to identify and subsequently follow the fate of candidate CSCs. Levings et al. (2009) generated a transgenic osteosarcoma cell line expressing a green fluorescent protein (GFP) reporter gene under the control of the stem cell-associated gene *OCT-4*. Subsequent enrichment of GFP+ cells yielded a population with increased tumor formation and metastatic capacity compared to GFP-depleted cells, and gave rise to heterogeneous tumors suggestive. However, it is noteworthy to mention that GFP-depleted cell populations were also capable of forming tumors in NOD/SCID mice albeit at higher concentrations. Nevertheless, genetic lineage tracking of cells would be an extremely valuable tool for the prospective identification and tracking of candidate sarcoma CSCs during its evaluation for stem cell properties.

Observations of elevated ALDH activity in normal tissue stem cells, as well as in hematopoietic and solid tumor CSCs, have led to the use of ALDH activity as a means of identifying CSC populations in sarcomas. Wang et al. (2011b) examined osteosarcoma cell lines and found that all cell lines contained a sub population of cells with high ALDH activity (ALDH^Br^) and which possessed increased tumorigenic capacity compared to ALDH^Low^ cells, increased proliferative and clonogenic abilities, showed increased expression of stem cell markers, and self-renewal capacity. In contrast, ALDH^Low^ cells were incapable of forming tumors in NOD/SCID mice. In a follow-up study, pre-treatment of ALDH^Br^ cells with bone morphogenetic protein-2 (*BMP-2*), which belong to a class of molecules known to be important in bone and cartilage formation, can effectively inhibit the tumor-initiating properties of ALDH^Br^ cells (Wang et al., 2011a). These results support the use of ALDH^Br^ selection to identify and isolate a sarcoma CSC-like population from osteosarcoma cells and illustrate the potential utility of CSC-based research in identifying novel therapeutic targets (e.g., BMP-2 signaling pathway). However, in a similar study evaluating the ALDH^Br^ cells derived from the osteosarcoma cell line MG63, ALDH^Br^ as well as ALDH^Low^ cells equally showed the ability to reconstitute the original whole cell population putting into question the validity of ALDH activity as a specific sarcoma CSC marker (Honoki et al., 2010). Further testing in a broader panel of cell lines and primary tumors may clarify this issue. Additionally, the arbitrary cutoff for high and low ALDH expression may also contribute to differing results. Thus, similar to the issue with using SP as a means of identifying CSC populations, consensus definitions for these assays will be essential for acquiring consistent experimental outcomes and effective translation of data among different investigators.

### Ewing sarcoma

Ewing sarcoma is the second most common malignant tumor of bone and soft tissue in children and young adults. Ewing sarcoma comprises a group of tumors with a spectrum of phenotypic and clinical manifestations and is characterized by the presence of unique fusion oncoproteins which join the *EWSR1* gene with several members of the *ETS* family of transcription factors, most commonly *FLI-1* (∼80% of clinical cases) (Balamuth and Womer, 2010). Although the exact functions of these fusion oncoproteins remain to be more clearly elucidated, expression of the fusion oncogenes is essential to the oncogenic phenotype of Ewing sarcoma as well as in the regulation of genes involved in metastasis and chemoresistance (May et al., 1993; Lessnick et al., 1995; Denny, 1996; Thompson et al., 1999; Arvand and Denny, 2001). Similar to osteosarcomas, survival in the metastatic or recurrent disease setting remain abysmally low at<20%. If sarcoma CSCs do indeed exist for Ewing sarcoma and are responsible for the resistance to conventional chemotherapeutic interventions, the identification and selective targeting of the CSC subpopulation will be essential in securing durable curative responses.

Similar in approach to the identification of CSC subpopulations in osteosarcoma cells, Suva et al. (2009) were the first to report on the isolation of CD133+ cells derived from primary Ewing sarcoma tumors. They showed that CD133+ cells were capable of initiating and forming tumors in NOD/SCID mice and were capable of recapitulating the parental tumor phenotype upon serial transplantation in NOD/SCID mice. Furthermore, only the CD133+ subpopulation could renew both CD133+ and CD133− cell populations. In a subsequent study by the same group, Riggi et al. (2010) expressed the characteristic Ewing sarcoma fusion oncogene *EWS/FLI-1* in undifferentiated human MSCs (hMSC^EWS/FLI-1^) derived from pediatric bone marrow samples and demonstrated the generation of a CD133+ subpopulation of cells with stem cell properties. However, CD133+ cells derived from hMSC^EWS/FLI-1^ were unable to form tumors in NOD/SCID mice suggesting that *EWS/FLI-1* expression in human MSCs is insufficient for tumorigenesis and despite the change in phenotype reminiscent of stem-like cells.

In contrast to the previously described work, Jiang et al. (2010) found heterogeneous expression of CD133 in 48 primary Ewing sarcoma tumors examined. Only 4 of the 48 Ewing sarcoma tumors exhibited high expression of CD133, and this heterogeneity in CD133 expression was similarly observed in nine Ewing sarcoma cell lines studied. With the exception of one cell line, STA-ET-8.2, no differences in chemoresistance or tumorigenic potential was observed between CD133+ and CD133− subpopulations. Thus, the significance of CD133+ in identifying a CSC population in Ewing sarcoma remains to be seen. Wahl et al. (2010) demonstrated that Ewing sarcoma cells expressing the neural crest marker CD57 (HNK-1) identified a population of highly tumorigenic cells with features of a CSC including self-renewal abilities on repeat serial transplantation and multipotent differentiation capacity in the CD57^High^ cell fraction. Interestingly, the tumorigenic CD57^High^ fraction did not consistently correlate with the coexpression of CD133 and CD57^High^ cells absent CD133 expression were highly tumorigenic suggesting that other cell surface markers can similarly be utilized to identify cells with CSC properties. It is important to point out that CD57^Low^ cells were also capable of inducing tumor formation and maintain this ability with serial transplantation, albeit at a much lower capacity, precluding the use of cell surface markers such as CD57 or CD133 in identifying the stem cell population in Ewing sarcoma.

The utility of SP cells in identifying stem-like cells in Ewing sarcoma has also been explored. Yang et al. (2010) isolated the SP fraction from the Ewing sarcoma cell line SK-ES-1 and showed that the SP cells can give rise to both SP and non-SP cells. SP cells also exhibited increased clonogenicity, invasive behavior, and cytotoxic drug resistance when compared to parental and non-SP cells. However, the ability of the SP population to propagate tumors *in vivo* was not performed. SP cells have also been observed in other Ewing sarcoma cell lines (Komuro et al., 2007), but prospective validation of this cell population through serial transplantation experiments would need to be performed to determine the presence of putative sarcoma CSCs.

Isolation of cells with high ALDH activity has also shown promise in identifying cell populations in Ewing sarcoma enriched for cells with potent tumor-initiating ability. Awad et al. (2010) examined Ewing sarcoma cell lines and patient-derived Ewing sarcoma xenograft tumors and defined an ALDH^High^ and ALDH^Low^ population (defined as cells with the highest and lowest 2% ALDH activity). Cells from the ALDH^High^ sub-population demonstrated CSC properties and were highly tumorigenic. ALDH^High^ cells were also more resistant to the cytotoxic effects of chemotherapy drugs. Interestingly, a selective inhibitor of *EWS/FLI-1* (YK-4-279) was effective in inhibiting the growth and tumorigenic capacity of both ALDH^High^ and ALDH^Low^ cells indicating the dependence of the ALDH^High^ population on *EWS/FLI-1* oncogene expression (Awad et al., 2010). Furthermore, the investigators compared the clonogenic and tumorigenic properties of ALDH^High^ cells compared to a cell population enriched for CD133+ cells and demonstrated superior colony formation and tumor induction when using cells derived from the ALDH^High^ fraction suggesting superior enrichment of putative CSCs using the ALDEFLUOR-based assay. However, as previously mentioned, the delimitation of ALDH^High^ or SP fractions are arbitrary and differ among investigators, and this lack of specific consensus and guidelines will hinder the prospective analysis of data utilizing these techniques.

### Rhabdomyosarcoma

Rhabdomyosarcoma (RMS) is the most common soft tissue sarcoma and the third most common extracranial solid tumor encountered in children while being exceedingly rare in adults. Similar to Ewing sarcoma, ∼20–30% of RMS cases involve the presence characteristic chromosomal translocations resulting in the production of an aberrant fusion oncogene. The *PAX3/FOXO1* (previously designated *PAX3/FKHR*) and the less common *PAX7/FOXO1* have classically characterized a more clinically aggressive subset of RMS referred to as the alveolar variant. The more common embryonal RMS variant is not associated with any characteristic cytogenetic abnormalities and tends to have a more favorable prognosis. Also similar to both osteosarcomas and Ewing sarcoma, RMS involves multi-modality treatment with generally favorable survival outcomes in the localized disease setting (∼70–80% survival) and poor outcomes for metastatic disease (∼20% survival).

There remains little published work regarding the determination of a CSC-like population in RMS. Komuro et al. (2007) showed that RMS, along with Ewing sarcoma and neuroblastoma cells possessed a SP of cells based on Hoechst 33342 dye exclusion. More recently, Walter et al. (2011) generated spherical colonies (termed rhabdospheres) from five RMS cell lines and demonstrated that these cells possessed stem cell properties including elevated expression of stem cell markers (*OCT-4*, *NANOG*, *c-MYC*, *SOX2*, and *PAX3*) and formed tumors at lower cell densities compared to adherent cells. The investigators further sorted the cells derived from spherical colonies and isolated a CD133+ cell fraction. The CD133+ cells, in contrast to CD133− and unsorted cells, were able to generate tumors in NOD/SCID mice at lower cell densities and were more resistant to treatment with the chemotherapy drugs cisplatin and chlorambucil. Furthermore, analysis of CD133+ expression in patient-derived tissue samples revealed that high CD133+ expression correlated with inferior survival compared to low or intermediate expression levels in patients with the embryonal RMS variant. Hence, CD133 expression in RMS may not only be useful in enriching for candidate sarcoma CSCs, but may also have clinical utility in predicting survival outcomes. It remains to be seen if selection based on ALDH activity, perhaps in conjunction with CD133 expression, would further enrich for cells with stem cell-like properties.

### Other soft tissue sarcomas

Given the identification and isolation of cells with stem cell-like properties in osteosarcoma, Ewing sarcoma, and RMS, there has been increased interest in the search for similar stem-like cells in other sarcoma subtypes. Stratford et al. (2011) have identified candidate sarcoma CSCs in a liposarcoma cell line (SW872) after double enrichment for CD133 and ALDH^High^ activity (ALDH^High^). Cells doubly positive for CD133 and ALDH^High^ were more clonogenic and tumorigenic when compared to parental cells or cells singly positive for either CD133 or ALDH^High^. Similarly, chondrosarcoma cells derived from primary tumors and enriched for CD133 also showed potent tumorigenic potential (Tirino et al., 2011). Synovial sarcoma tumors and cell lines also express CD133, though it remains to be shown whether these cells possess stem cell-like properties and are tumorigenic (Terry and Nielsen, 2010). However, a SP of cells derived from a primary synovial sarcoma tumor, as well as from other mesenchymal solid tumors (e.g., malignant fibrous histiocytoma) were capable of forming tumors in NOD/SCID mice in one investigation (Wu et al., 2007). However, it is unclear if the SP cells isolated in the latter study necessarily represent a population of cells with CSC properties or cells selected for heightened proliferative and tumorigenic capacity since SP and non-SP cells formed tumors.

## Conclusion

The search for the elusive CSC in sarcomas and other solid tumors remains a challenging and controversial area of research. The immense clinical and biological diversity of sarcomas add to the difficulties in performing research within this family of malignant tumors. Additionally, if one considers that the search for the sarcoma CSC involves a search for cells that may exhibit varying degrees of “stemness” dependent upon microenvironmental cues as recent research suggests (Chaffer et al., 2011), the challenge of CSC research in sarcomas appears daunting given current technologies.

Work in normal tissue stem cells and CSCs derived from other solid tumors have expanded our current understanding of both normal and malignant stem cell biology and have led to the development of novel techniques to identify and study the CSC population within a heterogeneous tumor. In this review, we have discussed the data with regards to the promise of cell surface markers such as CD133, CD117, or Stro-1 in the identification of candidate sarcoma CSCs. Refinements in flow cytometric techniques have allowed for the identification of SPs of cells which exhibit stem cell-like properties including self-renewal, tumor initiation and serial propagation, and inherent resistance to cytotoxic chemotherapy agents. At the very least, the techniques and assays described have allowed for the isolation of cell populations that contain within it the ever elusive CSC. Further technical refinements and use of novel techniques such as genetic lineage tracking may someday allow for the identification and isolation of a more pure population of CSCs from sarcomas – a goal yet to be achieved. In light of recent work suggesting the ability of a CSC to interconvert between tumorigenic and non-tumorigenic states depending upon tumor microenvironmental cues (Quintana et al., 2008; Chaffer et al., 2011), improved understanding into the cell origins of sarcomas and further refinements in the molecular characterization of sarcoma subtypes will be essential in the discrimination of putative CSCs contained within any given tumor. Furthermore, establishment of consensus definitions of what constitutes a CSC and more defined criteria for the delineation of candidate stem cells identified via the various assays described above (e.g., SPs, ALDH^High/Bright^ populations) will be paramount to any progress achieved in the study of stem cell biology. In the future, the unequivocal identification and validation of the existence of CSCs in sarcomas, or any other cancer, coupled with the development of drugs that will specifically target this population, may allow for a paradigm shift in how we view the evolution of cancers, as well as how we develop future strategies for effectively eradicating cancers in children and adults.

## Conflict of Interest Statement

The authors declare that the research was conducted in the absence of any commercial or financial relationships that could be construed as a potential conflict of interest.
